# Biogenically Synthesized Polysaccharides-Capped Silver Nanoparticles: Immunomodulatory and Antibacterial Potentialities Against Resistant *Pseudomonas aeruginosa*

**DOI:** 10.3389/fbioe.2020.00643

**Published:** 2020-07-21

**Authors:** Nehal M. El-Deeb, Mai A. Abo-Eleneen, Lamiaa A. Al-Madboly, Mona M. Sharaf, Sarah S. Othman, Omar M. Ibrahim, Mohammad S. Mubarak

**Affiliations:** ^1^Biopharmacetical Products Research Department, Genetic Engineering and Biotechnology Research Institute, City of Scientific Research and Technological Applications, New Borg El-Arab City, Alexandria, Egypt; ^2^Department of Biology and Biotechnology Program, Indiana University, Bloomington, IN, United States; ^3^Microbiology Department, Faculty of Science, Tanta University, Tanta, Egypt; ^4^Department of Pharmaceutical Microbiology, Faculty of Pharmacy, Tanta University, Tanta, Egypt; ^5^Protein Research Department, Genetic Engineering and Biotechnology Research Institute, City of Scientific Research and Technological Applications, New Borg El-Arab City, Alexandria, Egypt; ^6^Pharmaceutical Bioproducts Research Department, Genetic Engineering and Biotechnology Research Institute, City of Scientific Research and Technological Applications, New Borg El-Arab City, Alexandria, Egypt; ^7^Department of Medicine and Translational Research, Roswell Park Comprehensive Cancer Center, Buffalo, NY, United States; ^8^Department of Chemistry, The University of Jordan, Amman, Jordan

**Keywords:** green synthesis, AgNPs, anti-inflammatory, antibacterial, antibiofilm, virulence, wound healing

## Abstract

Bacterial infections are the key cause of death in patients suffering from burns and diabetic wounds while the use of traditional antibiotics has been growing steadily. Thus, in the present study, we are trying to introduce a paradigm shift strategy to improve chronic wound healing of bacterial infection. To that end, we have biologically synthesized silver nanoparticles (AgNPs) using *Arthrospira sp* polysaccharides, and evaluated their antibacterial efficacy with their safety pattern. Scanning electron micrographs showed spherical AgNPs coated with algal polysaccharides with an approximate size of 9.7 nm. Treatment of *Pseudomonas aeruginosa* with the AgNPs (0.5–1 μg/mL) resulted in a significant disruption in *P. aeruginosa* outer membrane, reduction in biofilm formation, and a significant decrease of production of alginate and pyocyanin along with a concentration-dependent reduction in β-lactamase activity. In addition, at the *in vivo* level, AgNPs displayed substantial activity to control *P. aeruginosa* infections in rat skin wounds with significant reduction in in COX-2 enzyme in both rat skin homogenate and serum samples. Furthermore, AgNPs facilitated wound curative in the *P. aeruginosa* infected model by reducing the hemorrhagic areas number and the infiltrated inflammatory cells. Taken all together, these biogenic nanoparticles showed unique properties in controlling bacterial wound infections and improving the healing process of damaged tissues via its direct and indirect effects.

**Graphical Abstract:**
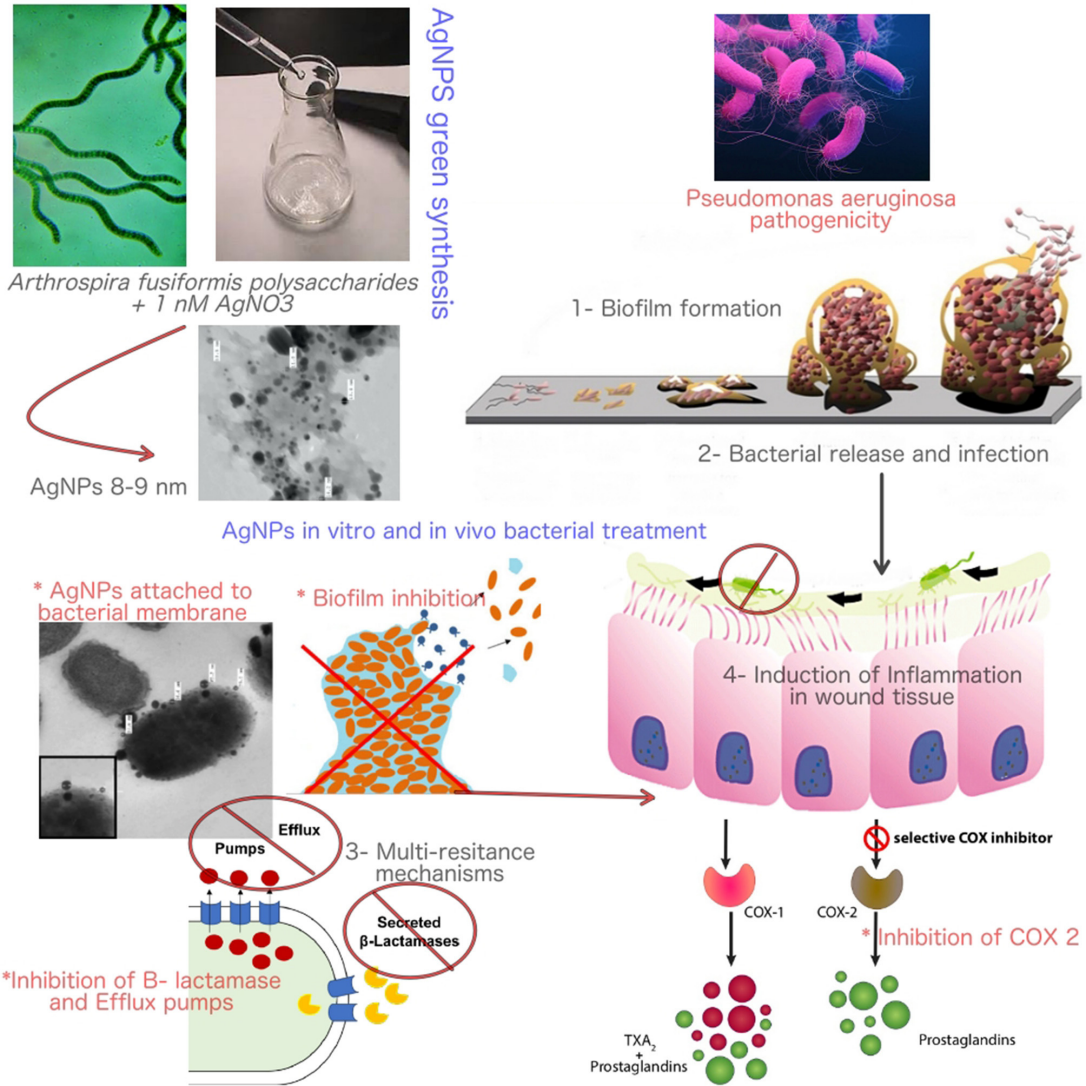
The mode of action of Bio-AgNPs in controlling *Pseudomonas aeruginosa* infections and pathogenicity.

## Introduction

Nanomaterials have attracted considerable attention owing to their potential use as drug delivery vehicles which found applications in cancer therapy. In this context, medical usage of these materials is dictated by the interaction of these nanoparticles and various human cells and tissues (Croissant et al., 2016). They have been employed as delivery systems in cases of poor solubility of drugs, and in the case of extensive side effect (Ashe, 2011). Nanotechnology and nanomedicine started a new era in targeted and specific therapy. Use of these very small, tiny, and specific particles has increased, as new vehicles for drug delivery, and also as effective drugs, in cases where toxicity and emergence of drug-resistance pathogens limit the use of some drugs. Size of these particles ranges from 1 to 100 nm (Croissant et al., 2016).

Due to its nano-scale size, nanoparticles display new, and significantly enhanced physico-chemical, and biological characters. This nano-scale size usually provides larger surface areas to nanoparticles (NPs) over the macro-sized particles (Jain, 2000). Numerous techniques have been successfully used to prepare nanoparticles which can be classified as (i) polymers-including methods and (ii) methods involving monomers polymerization. These approaches involve, but not limited to, (a) emulsion–solvent evaporation, (b) processing of salting out, (c) using supercritical fluid in production, (d) *in situ* polymerization and phase separation (Hakim et al., 2005), and (e) the biological approach which includes plant or animal extracts with different types of microorganisms such as bacteria, algae, and fungi which have been used to synthesize different metallic nanoparticles (NPs). This biological method has numerous advantages over the previously mentioned chemical methods as it saves more energy and is cost effective. The biologically synthesized NPs coated with biological molecules on its surfaces makes them biocompatible in comparison with the chemically synthesized NPs (Mishra et al., 2012). In this regard, *Penicillium rugulosum*, the important fungus in the industrial process, was used in the synthesis of a uniform sized Au NPs, which are easier to use as compared with those synthesized by bacteria and yeast (Patel et al., 2015).

Among the various biological systems used in the synthesis of NPs, many algal species have been recently employed. Also, these are more successful in the diverse of metal and metal oxide NPs fabrications (Philip, 2009). In this regard, the spherical AuNPs with sizes ~15 nm, biologically synthesized by natural honey have been used as a reducing and stabilizing agent. The fructose sugar in the honey, a reducing agent, and the honey proteins were responsible for stabilization of the NPs (Kannan et al., 2013). Furthermore, silver nitrate reduction was achieved by flavonoids and terpenoids metabolites of seaweed *Chaetomorpha linum* extracts as these metabolites acted as the capping and stabilizing agents during NPs development. These recovered NPs formed with an average size of 30 nm and showed potential applications in the medical fields (El-Rafie et al., 2013). In addition, various marine algal species including *Pterocladia, capillacae, Jania rubins, Ulva faciata*, and *Colpmenia sinusa* are used to produce AgNPs (Sabbah et al., 2018). Previous report of Cepoi et al. indicated that, *Spirulina Platensis* is an ideal selection for NPs bio-fabrication because the cells have many bioactive reducing compounds (Cepoi et al., 2015). *Arthrospira* sp extracts have been reported to exhibit diverse activity as anticoagulant (Majdoub et al., 2009) and antiviral effects (Chirasuwan et al., 2007).

Resistance to antimicrobial drugs is a threat to public health and is growing at an alarming rate. In the United States, statistics revealed that the number of deaths caused by the multi-resistant species, Methicillin-resistant *Staphylococcus aureus* (MRSA) is more than those caused by AIDS (Sharma et al., 2009). Therefore, there is a pressing need for the development of drugs to control and combat microbial resistance, whether in the form of new antibiotics or antimicrobial agents. In this context, various studies indicated the efficacy of silver nanoparticles against different bacterial strains (Mohanty et al., 2012). Silver nanoparticles strong antimicrobial effects have been shown in many applications as antibacterial textiles, medical devices coatings, home appliances product, and many cosmetics products due to its antibiofilm activities against different pathogenic bacteria such as *Salmonella, Pseudomonas, Staphylococcus*, and *E. coli* (Singh et al., 2018). This activity against bacterial biofilm has been broadly recognized (Markowska et al., 2013), especially, these activities by plant or microbial biosynthesized nanoparticles (Thuptimdang et al., 2015; Singh et al., 2018; Ahmad et al., 2020). In spite of all published reports that proved the AgNPs antibacterial and antibiofilm actions, the silver nanoparticles antibacterial mechanisms were not fully investigated, and the fact that they act against bacteria is not fully explained (Yassin et al., 2017). Accordingly, in the present study we have synthesized and characterized new silver nanoparticles (AgNPs) using the green synthetic approach and evaluated their antibacterial activity against six different antibiotic-resistant pathogenic bacterial strains. In addition, we have examined the mechanism of action at both *in vitro* and *in vivo* levels.

## Materials and Methods

### Materials

Silver nitrate (AgNO_3_; Sigma-Aldrich) and cell culture media (Lonza) where obtained from commercial sources, while microbial strains were clinically isolated, cultured, and tested for antibiotic resistance at the biopharmaceutical laboratory, SRTA, Alexandria, Egypt. *Arthrospira* sp was cultured on a modified Zarrouk medium at ~ 25 ± 2°C. All used cell lines were acquired from ATCC. *E. coli* K12 AG100 and *E. coli* HB101 were obtained from the Department of Pharmaceutical Microbiology, Faculty of Pharmacy, Tanta University.

### Methods

#### Preparation and Culturing of Microorganisms

*Staphylococcus aureus, Streptococcus mutans, Pseudomonas aeruginosa, Salmonella enterica, Escherishia coli*, and *Proteus sp*. (antibiotic resistant bacterial strains) were acquired from the culture collection of Genetic Engineering and Biotechnology Research Institute, Egypt. All bacterial isolates were sub-cultured in Luria Bertani broth media overnight at 37°C. Similarly, *Arthrospira fusiformis* was cultured on a modified Zarrouk medium at 31 ± 1°C for 4 days at the Biopharmaceutical Product Research Department, Genetic Engineering and Biotechnology Research Institute. Approximately 30 mL of *Arthrospira* pre-grown culture was inoculated to about 100 mL of modified Zarrouk medium. Algal cultures were illuminated using constant cool white fluorescent light (2500 LUX) with stirring to keep them homogenous. After incubation time, the culture cell free supernatant was recovered by centrifugation at 1792 RCF for 30 min at 4°C, and the recovered filtrate was filtered by means of a Whatman filter paper to remove any suspended algal mass. Polysaccharide sample extracted by ethanol precipitation method.

#### Characterization of Biogenic AgNPs and Their Safety Pattern With PBMCs

##### Green synthesis of AgNPs

Approximately 200 mL of *A. fusiformis* polysaccharide solution (2 gm/100 mL) was mixed, with shaking (220 rpm), with silver nitrate (AgNO_3_) to a final concentration of 1 mM for 1 h at room temperature. Silver nanoparticles (AgNPs) were recovered by centrifugation for 20 min at 9449 RCF, washed three times using phosphate buffer saline (PBS; 25 mM, pH:7.6), re-suspended in of PBS (25 mM, pH:7.6), and kept at 4°C (Yassin et al., 2017).

##### Characterization of AgNPs

*X-ray diffraction (XRD)*. Nanoparticles samples were collected by centrifugation at 8964 RCF for 30 min and the formed pellets were washed three times with distilled water. Then, the collected amounts were dried at 50°C for 16 h. Silver nanoparticles crystalline phases content was studied with the aid of X-ray diffraction (XRD- 7000, Shimadzu, Japan) with diffraction angle (2O) at a current of 30 mA, and 30 KV voltage with Cu Kα radiation (λ = 0.15418 A) in a range from 20 to 140 (20 range). The measurement was performed three times.

*Transmission electron microscopy (TEM)*. Nanoparticles samples were collected by centrifugation at 8964 RCF for 30 min, the formed pellets were dispersed in distilled water, and then sonicated to remove any aggregations. Microstructures of the biogenic AgNPs were examined with a Joel 6360LA transmission electron microscope (JEOL Ltd., Tokyo, Japan). Using double sided adhesive tape, the recovered AgNPs were prepared by placing 5 μL of the colloid solution on a carbon coated 3-mm copper grid, making a thin film of sample on the grid and extra sample was removed by filter paper and dried at room temperature. ImageJ software was used for analysis and poly dispersity index was calculated according to the following equation (El-Aassar et al., 2020; Ibrahim et al., 2020):

(1) $$\begin{array}{l} Poly\;Dispersity\;Index\;\left(PDI\right)={\left(\frac{Standard\;Deviation}{Mean\;Diameter}\right)}^{2} \end{array}$$

*AgNPs cytotoxicity assay; In vitro*. The nontoxic doses of AgNPs were determined on non-cancerous cells; (PBMC). Peripheral blood mononuclear cells (PBMCs) were isolated by gradient centrifugation according to the procedure outlined by Lohr and coworkers (Lomovskaya and Watkins, 2001). Cell suspension (6 × 10^4^ cells/mL) in RPMI media were cultured on 96-well plates and treated with 100 μL of each of extracts concentrations (to final concentrations of 400–12.5 μg/mL). After 48 h of incubation, AgNPs cytotoxicity percentages were measured using a neutral red assay (Borenfreund and Puerner, 1985). Healthy volunteers blood samples were collected, and all volunteers completed a written informed consent in agreement with our all Declaration. The protocols of blood sample collection were approved by the Research Ethical Committee at Faculty of Pharmacy, Egypt under international and institutional guidelines (REC-FPTU).

#### *In vitro* Antibacterial Assessment of AgNPs

This section describes the antibacterial effects of AgNPs against the multidrug resistant bacteria along with evaluation of their microbial biofilm inhibitory activity. Also, the effect of AgNPs on *Pseudomonas aeruginosa* ultrastructure and its multi-resistance mechanisms was evaluated.

##### Antibacterial assay for AgNPs

In order to assess the antibacterial effects of the newly synthesized AgNPs against the resistant tested bacterial strains, a microplate reader assay was carried out with some modifications (El-Deeb et al., 2015).

Briefly, about 100 μL of overnight bacterial cultures (10^4^ CFU/mL) were incubated with 100 μL of serially diluted AgNPs in LB broth at final concentrations of 250–0.37 μg/mL in 96 well plates; the inoculated plates were then incubated overnight at 37°C. The antibacterial % of AgNPs against the pathogenic bacteria was calculated according to the following equation:

(2) $$\begin{array}{l} Inhibition\;\%=\left(\frac{A-A1}{A0}\right)\;x\;100 \end{array}$$

Where each of A, A1, and A0 are is the absorbance of treated group, the blank, and the control group, respectively.

##### Biofilm quantification by crystal violet assay

Biofilm formation was measured in flat bottomed 96-wells microtiter plates by means of the crystal violet assay. The microbial over-night culture of all pathogens in LB medium were incubated at 37°C. Then, about 200 μL of bacterial suspension (10^4^ CFU/mL) was incubated with MIC concentration of AgNPs. The control group was adjusted as the untreated bacterial culture and LB media represented the blank control. After 24 h incubation, the loosely adhered culture was decanted, and the plates were gently thrice washed with sterile distilled water. The attached bacterial cells were stained for 15 min with 50 μL of crystal violet (0.1%). After rinsing twice, the cells bound dye was de-stained using 99% ethanol. The inhibition of microbial biofilm formation was quantified using a microplate reader by determining the dye intensity at 630 nm.

##### The effect of AgNPs on *P. aeruginosa* ultrastructure, virulence and multi-resistance mechanisms

*Transmission electron microscopy (TEM) of the treated Pseudomonas aeruginosa*. Approximately 20 mL of overnight *P. aeruginosa* cultures (6 × 10^4^ CFU/mL) was treated with AgNPs with a final concentration of 3.7 μg/mL for 60 min with (MIC concentration of AgNPs). The bacterial pellets were collected and washed three times with phosphate buffer saline (PBS; pH 7). Both treated and untreated bacterial pellets were cryofixed according to the method described by Bouhdid et al. (2009). The prepared ultra-thin sections were stained with 1% uranyl acetate and sodium citrate, and the microstructures of bacterial samples were observed by means of a Philips EM 301TEM microscope with a Pixel_calibration of 622.246 um and an Accel. Voltage of 120.0 kV.

*Pseudomonas aeruginosa virulence and resistance mechanisms: Effect of AgNPs on the pseudomonas pyocyanin production*. The effect of AgNPs on *pseudomonas* pyocyanin production was evaluated using the method of Prithiviraj et al. with some modification (Prithiviraj et al., 2005). Briefly, 10 mL of Luria Bertani (LB) broth was dispensed in sterile conical flasks followed by the addition of AgNPs at 1, 0.5, 0.25, and 0.125 μg/mL final concentrations. Overnight culture of *P. aeruginosa* was used to inoculate LB broth to give a final bacterial count of 6 × 10^4^ cells/mL. Following overnight incubation at 37°C in a shaking incubator at 90 rpm, cell free supernatant was separated by centrifugation at 1792 RCF for 10 min. Pyocyanin was recovered from 10 mL of culture supernatant by adding 6 mL of chloroform followed by vigorous shaking; this turns the blue-green color of pyocyanin into blue. The organic layer was relocated to a clean tube with 3.2 mL of 1N HCl, whereas the aqueous layer (pink in color) was used to measure pyocyanin spectrophotometrically at 520 nm. To calculate the concentration of pyocyanin, the absorbance was multiplied by 17.07.

*Effect of AgNPs on Pseudomonas aeruginosa alginate production*. *P. aeruginosa* was cultured in LB with and without AgNPs (0.25–1 μg/mL) different concentrations. After 24 h static incubation at 37°C, the viscosity of the broth was visually examined using the string test (Mathee et al., 1999).

*Effect of AgNPs on the activity and productivity of P. aeruginosa β-lactamase enzyme*. **Preparation of crude β-lactamase extract** Overnight culture of *P. aeruginosa* isolate was centrifuged for 10 min at 1008 RCF to harvest the cells. The bacterial pellet was washed with phosphate buffered saline PBS (pH 7), and after several washings, bacterial pellets were re-suspended in 1.5 mL of PBS and disrupted for 4 × 30 s using an ultra-sonicator processor (Cole Parmer, USA) with 30 s intermediate cooling times. The supernatant was separated by centrifugation at 1008 RCF for 10 min and used as a crude enzyme extract.

*Determination of β-lactamase activity*. *P. aeruginosa* bacterial cells were grown in LB broth with or without AgNPs (0.25, 0.5, and 1 μg/mL) until logarithmic phase was reached. Cells pellets were harvested, and intracellular extracts were prepared as described above. The enzyme activity was spectrophotometrically determined by nitrocefin substrate according to the procedure of O'Callaghan et al. (1972). One unit of β-lactamase is defined as the enzyme amount required to hydrolyze 1.0 μmole of nitrocefin per 1 min at 25°C and pH 7.0.

*Effect of AgNPs on the productivity of β-lactmase enzyme*. Luria Bertani broth was used to subculture *P. aeruginosa* isolate in the absence or presence of AgNPs at final concentrations of 0.125, 0.25, 0.5, and 1 μg/mL. After overnight incubation at 37°C in a shaking incubator, cell lysates were prepared as previously described. The amount of β-lactamase produced was expressed as activity, and the percentage of inhibited productivity of β-lactamase was calculated (O'Callaghan et al., 1972).

*Effect of AgNPs on the efflux pump of Pseudomonas aeruginosa isolate*. *P. aeruginosa* isolate was cultured in LB medium with or without AgNPs different concentrations and incubated at 37°C with agitation at 220 rpm until an optical density (OD) of 0.6 at 600 nm is reached. Cultures were OD adjusted with PBS to 0.5 by McFarland standard. *E. coli* K12 AG100, a positive control, was cultured in the presence of 10 mg/L of tetracycline. *E. coli* HB101 was also cultured in LB medium and used as a negative control. Trypton soy agar plates containing 1.5 mg/L of ethidium bromide were prepared; plates were divided into eight sectors by radial lines to give the cartwheel pattern. Bacterial cultures with adjusted OD were swabbed on the plates beginning with the center and directing into the edges. Plates were incubated for 16 h at 37°C followed by examination under UV transilluminator (Martins et al., 2011).

#### *In vivo* Evaluation of AgNPs Effects on *Pseudomonas aeruginosa* Infected Rats

This section will evaluate the efficiency of biogenic AgNPs in controlling *P. aeruginosa* wound infection in rat models. The evaluation scheme includes the histopathological examination, quantification of biochemical parameters, and monitoring of inflammatory regulators.

#### Ethical Statement

All *in-vivo* studies were carried out according to the guidelines of Tanta University, Faculty of Pharmacy, Tanta, Egypt (REC-FPTU). The Research Ethical Committee at the Faculty of Pharmacy, Egypt under international and institutional guidelines (REC-FPTU) approved all used experimental protocols.

##### Bacterial preparation and AgNPs ointment formulation

*P. aeruginosa* was cultured overnight in LB broth media using the previously mentioned conditions. At the end of incubation, bacterial cells were collected by centrifugation at 161 RCF for 20 min. These cells were washed three times with sterile saline, then the recovered cells were suspended in the sterile saline to bacterial count of 10^6^ CFU/mL saline. The tested ointment was prepared by mixing of 60 μg AgNPs (sub safe dose) with ~1 g of an ointment base contained cetosteryl alcohol, hard paraffin, wool fat, and white soft paraffin. The desired ointment base quantity was weighed and melted at 70°C in a water bath. The designated quantity of AgNPs was added to and stirred gently with the melted base at 40°C until a homogenous dispersion was obtained.

##### Wound creation

Twenty female Wistar rats (120–150 g) were used throughout this investigation. These animals were given free access to standard diet and water and were kept under standard conditions set according to the guidelines of the National Institute of Health (NIH). After 2 weeks of acclimation, the left dorsal thoracic region of the animal, 5 cm from the ear and 1 cm away from the vertebral column, was shaved and a 3-cm wound (diameter) was made. All tested animals were anesthetized with chloroform during the wound excision step. Animals were separated into four different groups with five rats each. The first group was used as the untreated, whereas the second represented the infected-untreated group: In this group, 1 day after wound creation, animal wounds were inoculated with 1 mL of the previously prepared of *P. aerugin*osa bacterial suspension (10^6^ CFU/mL) for 2 days in a 1-day daily dose. The third group was the infected-AgNPs—treated. In this group, 1 day after the last bacterial dose, each animal was treated with the formulated AgNPs ointment with a final concentration of 60 μg AgNPs/Kg animal body weight. Rats were inoculated with their respective doses every day for 7 days. The Last was the AgNPs-treated group, where animals received the same doses of AgNPs formulated ointment as mentioned in the third group with the exception that they were not infected with *P. aeruginosa*. Blood samples from sacrificed animals were collected and transferred immediately to ice. Blood plasma were recovered by centrifugation for 20 min at 1008 RCF and were stored at −80°C. Skin sections were removed and washed with chilled sterile saline solution. The tissue sections were singly minced and homogenized in ice-cold sodium and potassium phosphate buffer (0.01 M, pH 7.4). The cell debris free supernatants were recovered from tissue the homogenates by centrifugation at 1008 RCF for 20 min at 4°C, and the resultant supernatant was used.

##### Histological section preparation

Rat skin sections were collected and immediately fixed in 10% formalin, treated with alcohol and xylol, and embedded in paraffin. For studying the histopathological changes, the tissue sections were segmented at 4–6 cm thickness and stained with Haematoxylin and Eosin (H&E) stain (Putt, 1954).

##### Biochemical parameters

The total protein, albumin, urea, creatinine, and alkaline phosphatase were analyzed using the stored plasma samples. In addition, AST (aspartate transaminase) and ALT (alanine transaminase) enzymes were measured with commercial kits (BioSystems S.A. Costa Brava, 30. 08030 Barcelona, Spain).

##### The regulation of inflammatory mediators in Pseudomonas aeruginosa-infected animal model by AgNPs

*Quantification of cyclooxygenase 1 and 2 levels assay*. Levels of both cyclooxygenase one and two blood serum and skin homogenates were measured by ELISA kit specified for cyclooxygenase one and two protein concentrations (Cusabio, Biotech, wuhan, Hubei, China) in all tested animal groups. Blood sample sera of all groups were collected as previously mentioned, and the level of cyclooxygenase one and two enzyme concentrations were performed according to the manufacturer's instruction. Protein concentrations were read with a microplate reader at 450 nm. The cyclooxygenase 1, 2 enzyme concentrations was obtained from a standard curve.

*Quantification of the induced ROS in rat skin tissue isolated macrophages*. Skin isolated macrophages were performed according to a procedure described by Malosse and Henri with some modifications (Malosse and Henri, 2016). Briefly, at the end of treatment, mice were sacrificed. An area of ~1.5 × 1.5 cm^2^ of back naked skin was excised and the subcutaneous fat and blood vessels were carefully separated. Recovered skin samples were cut into small pieces and transferred to 1 mL of collagenase 4-DNase working solution and were incubated for 30 min at 37°C. After 30 min, cells were collected by centrifugation at 161 RCF for 10 min. Recovered cells were suspended in sterile PBS, and the desired ones were separated by means of percoll gradient centrifugation, as described by Lohr et al. (1995). Recovered cell rings at the interface were suspended in RPMI free serum culture media after lysis of RBCS using ACK lysis buffer. To remove the non-adherent T cells, the cell suspension was then incubated for 1 h. The recovered macrophage cells were washed with PBS and using the fluorescent membrane permeable probe 2,7-dichlorofluorescein diacetate (DCFH-DA) (Molecular Probes, Sigma Aldrich), The total induced ROS was measured by flow cytometry according to a published procedure (Yassin et al., 2017).

*Quantification of IκBα, IL-1α, and IL-1β, and TNF-α in rat skin tissues*. At the end of the *in vivo* study, the total animal skin RNAs were extracted using an RNA extraction kit (Thermo scientific). The current study focused on the expression levels of IL-1α (Interleukin 1 alpha), IL-1β (Interleukin 1 beta), TNF-α (tumor necrosis factor alpha), and IkappaB-α (kappa light polypeptide gene enhancer in B-cells inhibitor, alpha nuclear factor) genes. The first cDNA strands were synthesized using strand cDNA synthesis kit (Thermo scientific). Real time polymerase chain reaction (Rt-PCR) was conducted by Syber Green master mix (Qiagen) where GAPDH was used as an internal control reference. Used primers are listed in Table 1.

**Table 1:** The primers list used in gene expression analysis.

**Primers**	**Sequence**
IL1α-forward	5′- CAAGATGGCCAAAGTTCGTGAC 3′
IL1α-reverse	5′- GTCTCATGAAGTGAGCCATAGC 3′
IL1β-forward	5′- ATGGCAACTGTTCCTGAACTCAACT−3′
IL1β-reverse	5′- CAGGACAGGTATAGATTCTTTCCTTT−3′
TNFα-forward	5′- TTC TGT CTA CTG AAC TTC GGG GTG ATG GGT CC−3
TNFα-reverse	5′- GTA TGA GAT AGC AAA TCG GCT GAC GGT GTG G−3′
IkappaB-α forward	5′-CATGAAGAGAAGACACTGACCATGGAA-3′
IkappaB-α reverse	5′-TGGATAGAGGCTAAGTGTAGACACG-3′
β-actin forward	5′- GTGGGGCGCCCCAGGCACCA−3′
β-actin reverse	5′- CTCCTTAATGTCACGCACGATTTC-3'′

### Statistical Analysis

All determinations were conducted in triplicate and results are presented as the mean ± standard error of the mean (SEM). Data were subjected to one-way analysis of variance (ANOVA) with multiple comparisons, and two-way ANOVA with Tukey's test was used to assess comparisons between groups. Statistical analysis was performed with the aid of Student's *t*-test for significance with the aid of GraphPad Prism-8; differences were considered significant at *p* ≤ 0.05.

## Results

### Characterization and Safety Pattern of the Recovered AgNPs

**Figure 1:**
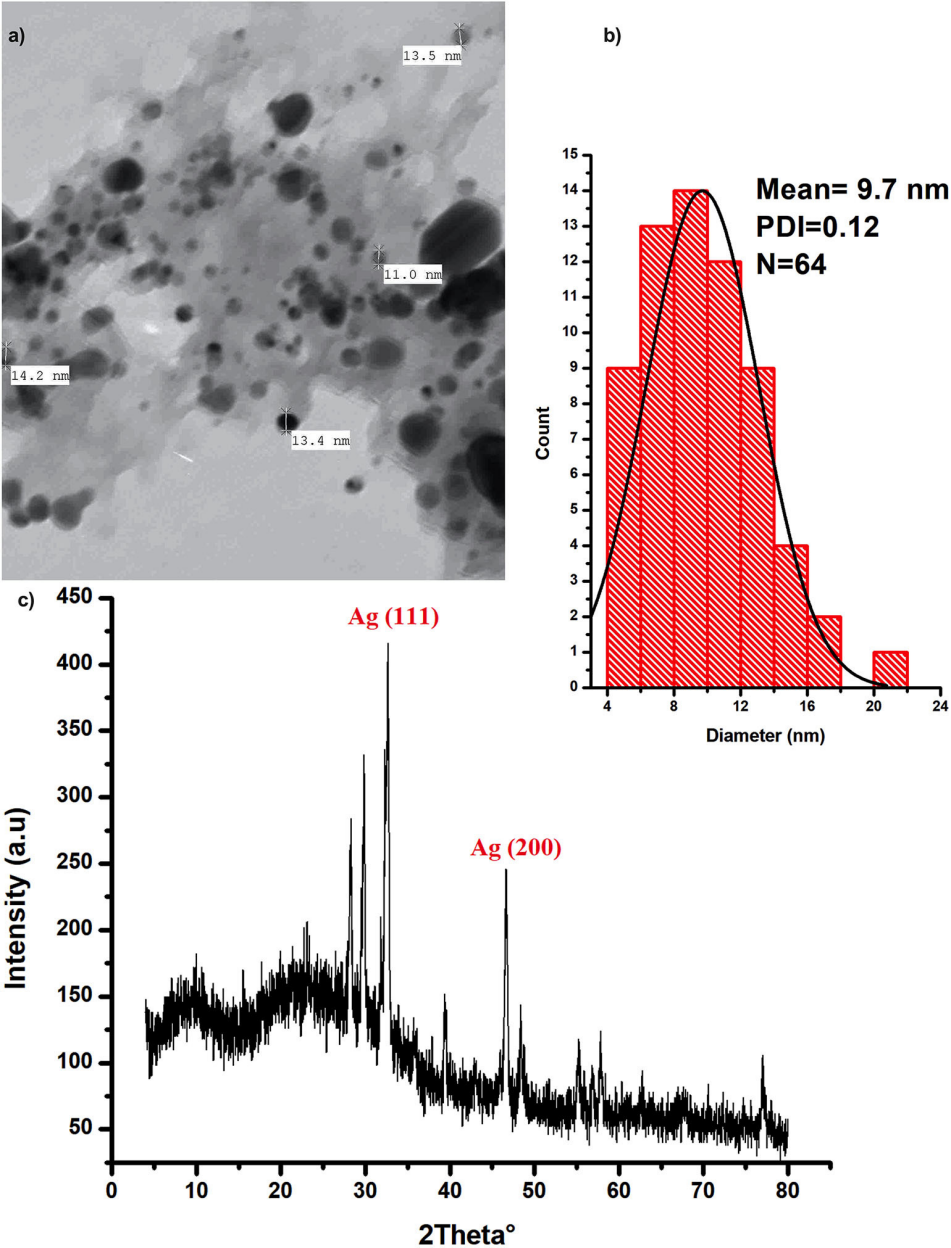
Characterization of the biogenic AgNPs; **(a)** the biosynthesized AgNPs SEM image **(b)** AgNPs particle size distribution **(c)** XRD spectrum of the dried powder of the biosynthesized AgNPs.

Silver nanoparticles were characterized with the aid of transmission electron microscopy and X-ray diffraction (XRD) analysis; (Figure 1). Data from these figures indicate that recovered biogenic AgNPs appear as spherical particles coated with bioactive ingredient of the extracted polysaccharides, with an approximate size of 9.7 nm and 0.15 poly dispersity index which indicates homogenous particulates distribution. XRD analysis shows development of the silver particles crystalline phases content in the biogenic AgNPs sample. The sharp peaks at 2θ of 32.639 and 46.612 are attributed to (111) and (200) crystallographic planes (El-Aassar et al., 2020; Ibrahim et al., 2020). In addition, the sample is characterized by Ag (111) peaks which shifted to a lower value of 32.639°, whereas the Ag (200) peak is shifted to a higher value of 46.612°, depending on the preparation conditions of the sample (Figure 1c).

**Figure 2:**
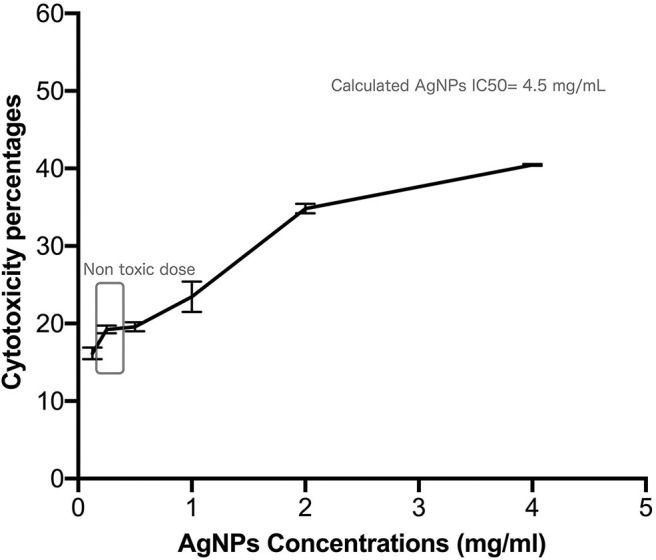
Safety assay pattern of the biosynthesized AgNPs on isolated peripheral blood mononuclear cells (PBMC). Different concentrations of AgNPs (final concentrations of 400–12.5 μg/mL) were incubated on PBMCs for 48 h. AgNPs cytotoxic effects were expressed as viability inhibition percentages of PBMC using neutral red assay compared with the untreated cells. All measurements were performed in triplicate and the average was calculated ± standard deviation (*SD*) using GraphPad prism8 software.

Safety pattern of biogenic AgNPs was tested on peripheral blood mononuclear cells (PBMC). Results indicated that an IC_50_ value of 453 μg/mL was obtained upon treatment of normal cells (Figure 2). The maximum used dose (4 mg/mL) showed cellular inhibition percentage of 40.5 and obtained nontoxic concentration on PBMC reached 70 μg/mL with 10% cellular inhibition.

### *In vitro* Antibacterial Assessment of AgNP

The antibacterial activity of biogenic AgNPs against different antibiotic resistant bacteria was measured by means of a microplate reader assay protocol, using different concentrations of AgNPs (3.7–500 μg/mL). Inhibition percentage of AgNPs treatment against bacterial growth ranged from 0.811 to 98.2% (Figures S1a,b); all tested strains were almost completely inhibited by using an AgNPs with a dose of 500 μg/mL. *Proteus* and *S. mutans* were the most sensitive bacterial strain to AgNPs among all tested strains at lower AgNPs concentrations. In addition, the calculated MIC values of AgNPs were 3.7 μg/mL against *S. aureus, P. aeruginosa*, and *S. enterica*, and 1.7 μg/mL for *S. mutans, E. coli*, and *Proteus sp*.

In addition, the positive effects of the biogenic AgNPs on biofilm formation of tested bacterial strains are shown in Figure S1c. Results reveal that treatments with AgNPs exhibit positive effects against biofilm formation of all tested strains with inhibition percentage ranging from 52.85 to 79.37%. Moreover, results show that *S. enterica* was the most sensitive strain to treatment without any significant difference with *S. mutans* that showed 76.61%. On the other hand, *P. aeruginosa* was the most resistant strain followed by *S. aureus* which showed 60.45% inhibition in biofilm formation upon treatment. Furthermore, both *Proteus sp*. and *E. coli* exhibited 71.42 and 69.11% inhibition, respectively, upon treatment with AgNPs.

### The Effect of AgNPs on *Pseudomonas aeruginosa* Ultrastructure, Virulence and Multi-Resistance Mechanisms

#### Ultrastructure Changes

**Figure 3:**
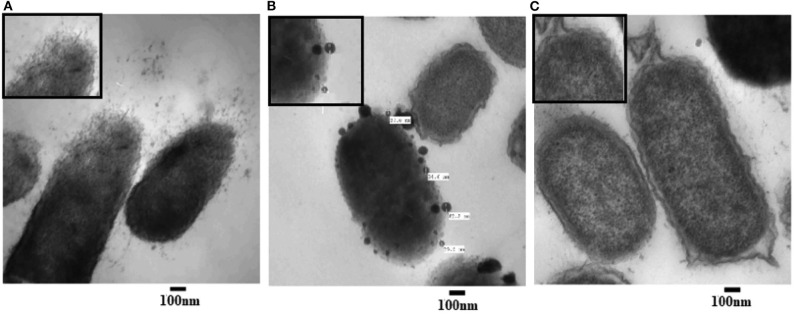
Transmission electron microscope (TEM) images of *P. aeruginosa* treated with AgNPs **(A,B)** and un-treated (**C**; control). Numerous disruptions of the cell wall were observed in the treated bacterial cells. AgNPs particles were detected near the outer cell's envelope in the treated cells. Intact bacterial membrane of the untreated cells.

To examine the morphological changes consequential to AgNPs treatment, TEM was achieved on tinny sections of treated (Figures 3A,B) and untreated *P. aeruginosa* bacterial samples (**Figure 7C**). Different morphological changes were observed upon treatment of *P. aeruginosa* cells with AgNPs (Figure 3). Several protrusions from the cell wall and membrane vesicles releasing were observed in the treated cells (Figure 3A). In addition, different AgNPs particles, close to the outer cell's envelope with disruptions in some regions of bacterial outer membrane, were observed inside the treated cells (Figure 3B). Moreover, in the neighboring areas, a significant amount of cytoplasmic material was noticed.

#### Virulence and Multi-Resistance Mechanisms

**Figure 4:**
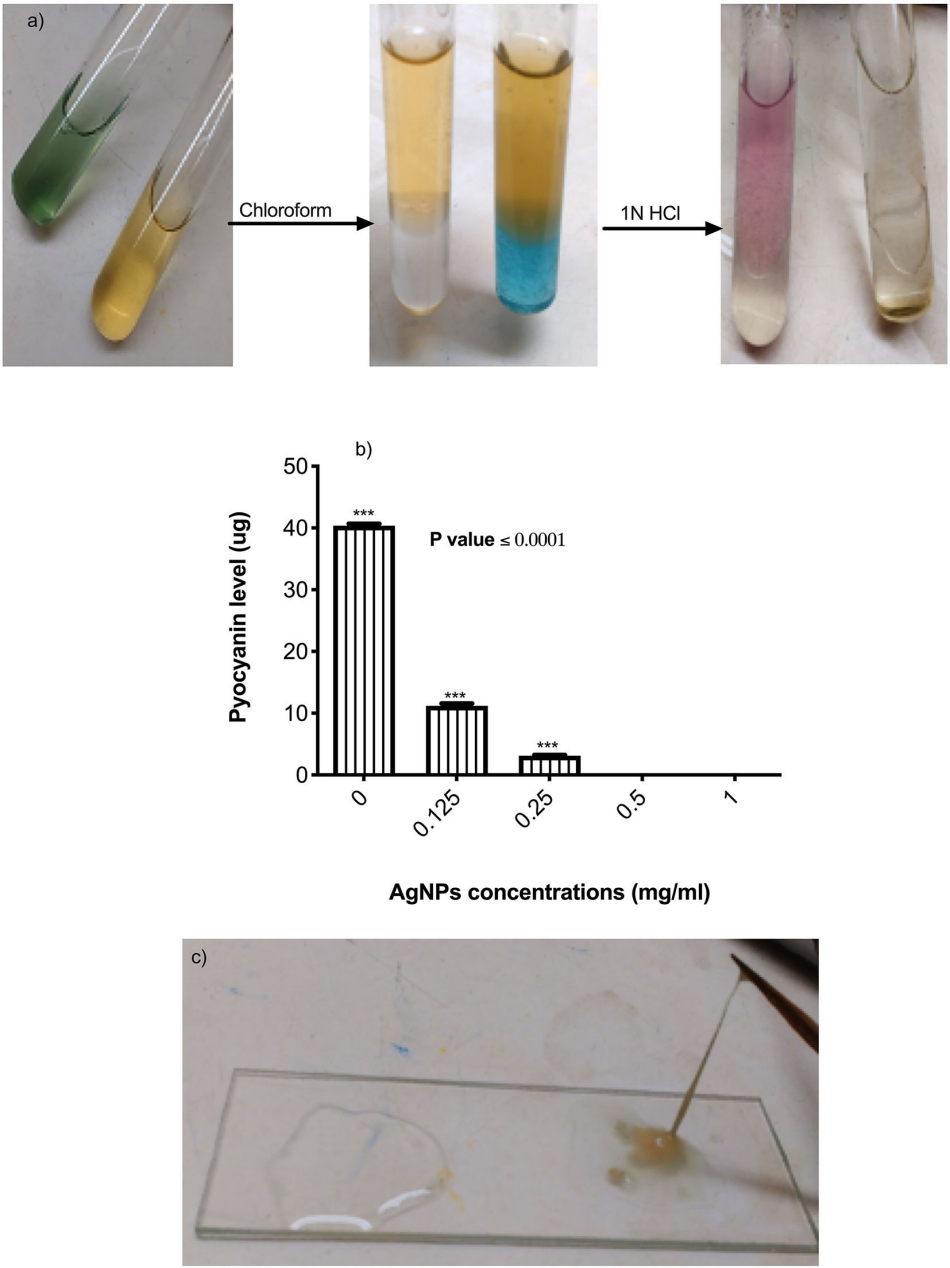
**(a)** Steps of pyocyanin extraction with or without 0.5 μg/mL of AgNPs. Complete inhibition in productivity was observed. **(b)** Quantification of pyocyanin levels of *P. aeruginosa* upon treatment with AgNPs. **(c)** Inhibition of alginate production (left side) by *P. aeruginosa* after treatment with 0.5 μg/mL of AgNPs. The negative control (right side) showed hyper-mucoviscosity by string test. ***is highly significant at *P* < 0.0001.

The effects of AgNPs on *P. aeruginosa* multi-resistance mechanisms were quantified, these mechanisms include *P. aeruginosa* production of alginate, pyocyanin, β-lactamase enzyme, and *p. aeruginosa* efflux pump. To study the effect of AgNPs on the production of pyocyanin, different sub (MICs) minimum inhibitory concentrations of AgNPs (0.125. 0.25, 0.5, and 1 μg/mL) were added to the bacterial growth media. Quantitative estimation of pyocyanin levels revealed a significant decrease in production compared to the control (Figures 4a,b). Percentage of reduction ranged between 98.25 and100 %, and the absolute percentage was achieved when 0.5 and 1 μg/mL was added to the growth media (Figure 4b). *P. aeruginosa* was grown with or without different concentrations of AgNPs. Following incubation, muco-viscosity of the broth culture was examined visually by the string test. Results revealed complete inhibition of alginate production at all tested concentrations of AgNPs compared to the negative control which showed highly viscous solution as observed in Figure 4c.

**Figure 5:**
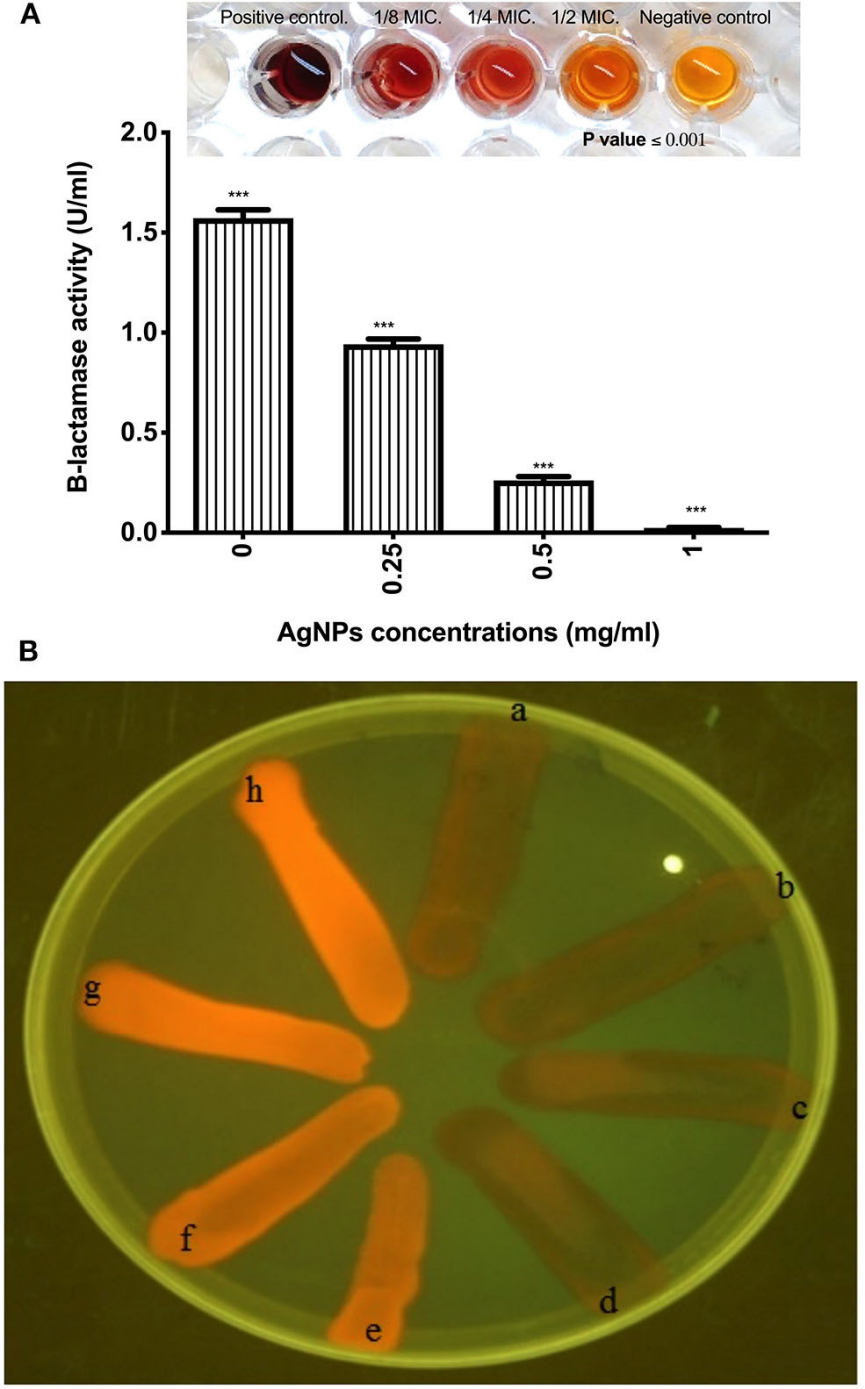
**(A)** Effect of AgNPs on the production of β-lactamase enzyme by *P. aeruginosa* isolate **(B)** Fluorescence of *P. aeruginosa* on ethidium bromide containing TSA plates using UV light. It shows a, Positive control *E. coli* K1 AG100; b, Untreated MDR *P. aeruginosa* isolate; c, *P. aeruginosa* isolate treated with 0.0625 μg/mL of AgNPs; d, *P. aeruginosa* isolate treated with 0.125 μg/mL of AgNPs; e, *P. aeruginosa* isolate treated with 0.25 μg/mL of AgNPs; f, *P. aeruginosa* isolate treated with 0.5 μg/mL of AgNPs; g, *P. aeruginosa* isolate treated with 1 μg/mL of AgNPs; h, *E. coli* HB101 Negative control. Fluorescence means negative efflux. ***is highly significant at *P* < 0.0001.

The effect of AgNPs with different concentrations on the activity and production of β-lactamase enzyme made by *P. aeruginosa* isolate was investigated. Results showed that silver nanoparticles markedly inhibited the production of the enzyme by the test isolate when treated with concentrations of 0.125. 0.25, 0.50, and 1.0 μg/mL as depicted in Figure 5A. Additionally, AgNPs at final concentrations of 0.25, 0.50, and 1.0 μg/mL reduced the activity of β-lactamase enzyme in a concentration-dependent manner as shown in Figure 5A. On the other hand, Cartwheel method was employed to assess the influence of different sub MICs of AgNPs on the efflux pump of *P. aeruginosa* (Martins et al., 2011). Results showed different range of degrees of fluorescent bacterial masses, depending on their abilities to efflux the substrate (ethidium bromide). Inhibited fluorescence means active efflux. This was observed when *P. aeruginosa* isolate was treated with 0.0625 and 0.125 μg/mL of AgNPs. Intermediate fluorescence was observed when the treatment concentrations were increased to 0.25 and 1.0 μg/mL which confirmed that the emitted fluorescence was concentration dependent as shown in Figure 5B.

### *In vivo* Evaluation of AgNPs Effects on *Pseudomonas aeruginosa*

#### Regulation of Induced Concentrations of Cyclooxygenase (COX) 1 and 2 in *Pseudomonas aeruginosa* Infected Rats by AgNPs

**Figure 6:**
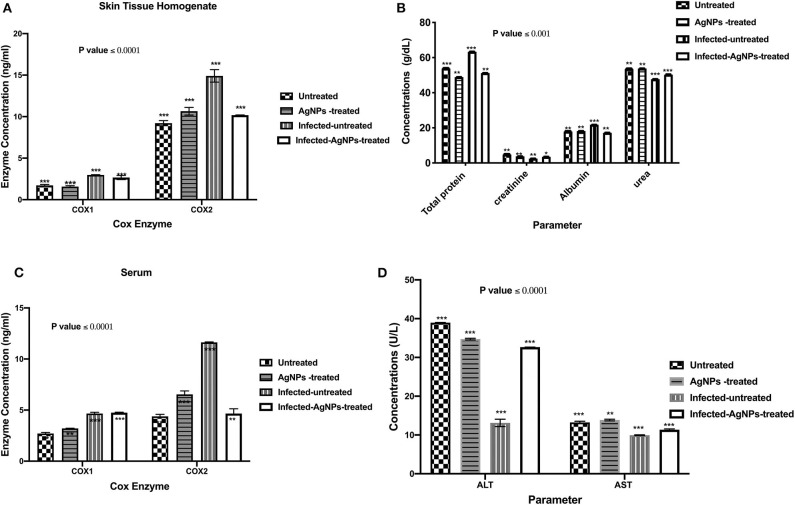
**(A)** The regulation of the induced COX-1&2 concentrations of the skin homogenates. AgNPs treatment decreased the elevated COX-2 concentrations from 14.38 (the infected group) to 10.16 ng/mL (the treated group) without a significant difference with the non-infected group. Also, in the serum samples **(B)** AgNPs treatment significantly decreased the concentration of the induced COX-2 from 11.66 (the infected group) to 4.34 ng/mL (the treated group). **(C)** Total protein, creatinine, albumin, and urea quantifications **(D)** ALT and AST concentrations, in *P. aeruginosa* infected rats treated with AgNPs. All measurements were done in triplicate and the average was calculated ± standard deviation (*SD*) using GraphPad prism8. Data are represented as the mean, standard deviation and *N* = 3, ***p < 0.0001, and **p < 0.0125 using two-way ANOVA. *is not significant at *P* < 0.0001. ***is highly significant at *P* < 0.0001.

ELISA was employed to measure the concentrations of COX-1 and the induced COX-2 enzymes in skin and serum samples of all used animal groups as depicted in Figures 6A,B. In rat skin homogenates (Figure 6A), no significant elevation in COX-1 concentrations among all tested groups was observed, whereas the infected-untreated rat skins showed a significant induction (14.38 ng/ mL) in COX-2 enzyme compared to untreated group (9.43 ng/ mL). However, upon treatment, the elevated concentrations of COX-2 enzyme decreased from 14.38 to 10.16 ng/ mL without a significant difference with the untreated group. On the other hand, after AgNPs treatment, no significant reduction in the serum elevated concentrations of COX-1 compared to the infected-untreated group was noticed (decreased from 4.78 to 4.75 ng/mL). Furthermore, in serum sample, AgNPs treatment significantly decreased the concentration of the induced COX-2 from 11.66 to 4.34 ng/mL).

#### Biochemical Parameters

Upon topical treatment with AgNPs (60 μg/ Kg BW), the total protein concentrations in serum samples of infected-AgNPs-treated groups significantly decreased from 36.2 g/dl in the infected-untreated group to 51.1 g/mL in AgNPs-treated group (*p* ≤ 0.0001), without significant differences from either AgNPs-treated and infected-untreated groups (53.8 and 48.9 g/dL, respectively) as shown in Figure 6C. On the other hand, creatinine concentrations dramatically decreased in the infected-untreated group (2.5 g/dL) with significant differences with other groups (*p* ≤ 0.0001). In addition, creatinine concentrations in both AgNPs-treated and infected-AgNPs -treated groups (3.8 and 3.5 g/dL, respectively) did not significantly differ from the untreated group (2.5 g/dL).

Similar results were obtained for urea and albumin. AgNPs did not show significant differences in both urea and albumin concentrations from the untreated group (53.8–53.5 and 18.3–17.9 g/dL, respectively). Furthermore, AgNPs treatment (infected-AgNPs-treated) significantly altered the concentrations of both urea and albumin compared with the infected-untreated group (from 50.2 to 47.7 and from 17.1 to 21.8 g/dL, respectively) (*p* ≤ 0.0001) (Figure 6C). On the other hand, bacterial infection dramatically decreased ALT and AST concentrations from 38.93 to 13.79 and from 13 to 10 U/L, respectively. However, upon treatment, both ALT and AST concentrations increased again to 32.65 and 11.5 U/L, respectively, without any significant differences with the untreated group. Moreover, AgNPs topical usage did not show any significant effects, *p* ≤ 0.0001, on the concentration pattern of both ALT and AST (34.57 and 13.7 U/L, respectively) (Figure 6D).

#### AgNPs Immunomodulatory Actions

**Figure 7:**
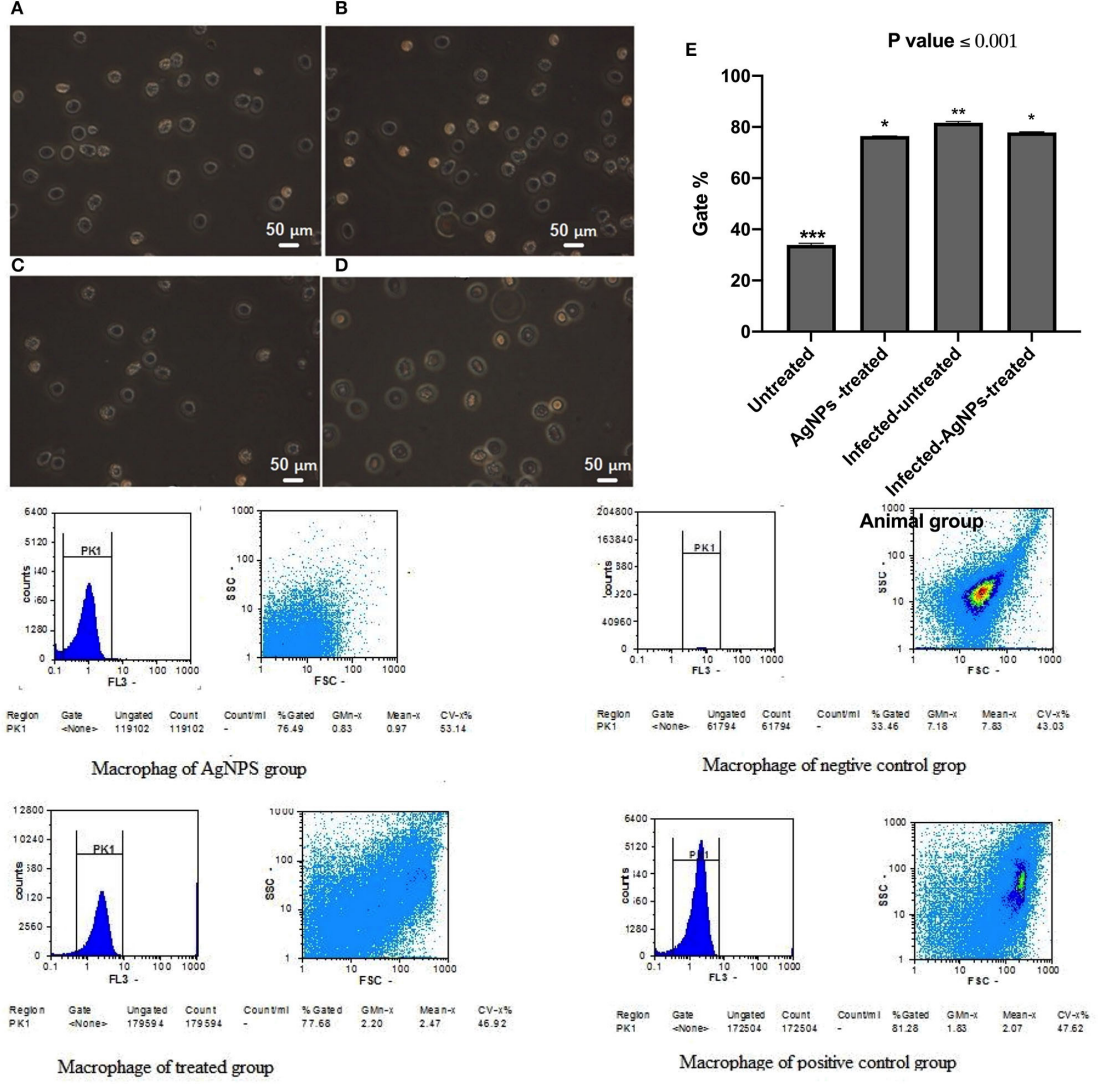
Isolated macrophages of rat skins where; **(A)** Rat skin isolated macrophages of AgNPs group (without bacterial infection). **(B)** Rat skin isolated macrophages of bacterial infected group (positive control). **(C)** Rat skin isolated macrophages of the non-infected group (negative control). **(D)** Rat skin isolated macrophages AgNPs treatment to the bacterial infected group (AgNPs -treated group). **(E)** Flow cytometric quantification of the induced ROS in in the *ex-vivo* skin associated macrophages of *P. aeruginosa* infected rats upon AgNPs treatments. *N* = 3. *is not significant at *P* < 0.001. **is significant at *P* < 0.001. ***is highly significant at *P* < 0.0001.

Immunomodulatory actions of AgNPs were monitored by measuring induced ROS in the *ex-vivo* skin associated macrophages (Figure 7) using flow cytometry. Results indicated that both *P. aeruginosa* bacterial infection and AgNPs treatment showed abilities to significantly induce intracellular ROS with potency to bacterial infection which induced ROS with gating percentage from 33.46 in untreated animal group cells to 81.27% in infected-AgNPs-treated animal group cells (Figure 7E). On the other hand, upon treatment, the gating percentage of induced ROS in infected-AgNPs-treated group significantly decreased from 81.28 to 77.68% without substantial differences with the AgNPs-treated group (76.49, *p* ≤ 0.0001).

**Figure 8:**
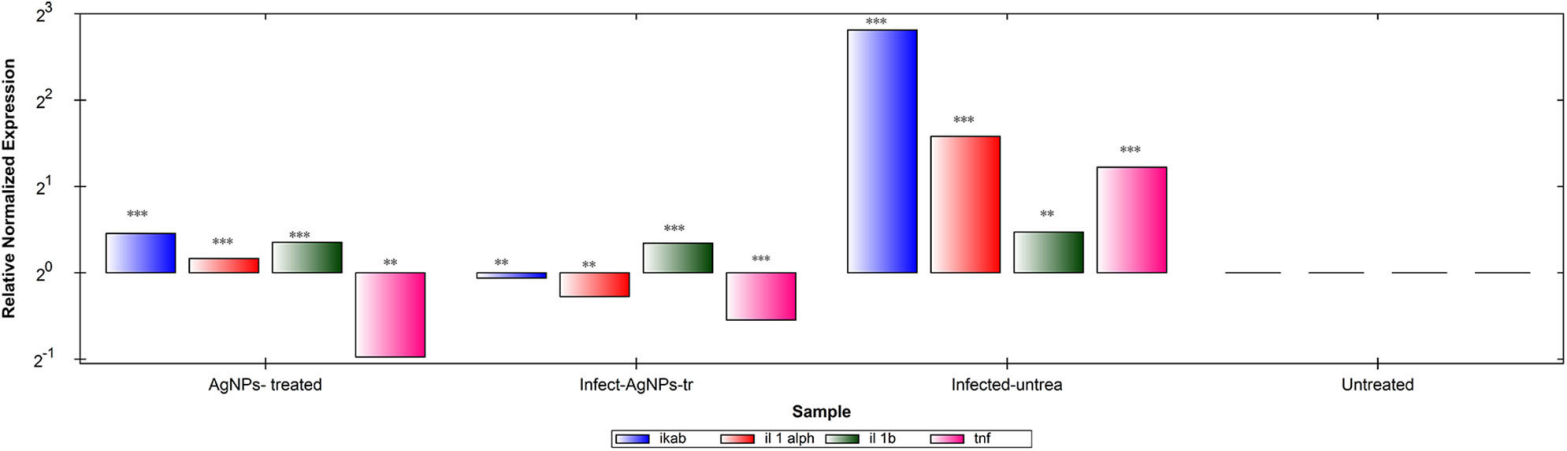
RT-qPCR analysis of the expression levels of IKaB, IL-1α, IL-1β, and TNF-α genes in rat skin samples upon AgNPs Treatments. The gene expression levels of IKaB (blue color), IL-1α (Red), IL-1β (green), and TNF-α (pink) with β-actin normalizations and compared to the control nine untreated cells). The gene levels below the control line represented the downregulation pattern and that above the control line represented the upregulated ones. All measurements were done in triplicate and the average was calculated ± standard deviation (*SD*) using the GraphPad prism8 software. **is significant at *P* < 0.001. ***is highly significant at *P* < 0.0001.

Gene expression patterns of IKaB, IL-1α, IL-1β, and TNF-α in rat skin samples were determined using RT-qPCR (Figure 8A). Upon normalization, using β-actin, *P. aeruginosa* bacterial infection showed abilities to up-regulate levels of expression of all tested genes in comparison with the negative control group. Upon treatment, AgNPs exhibited abilities to down-regulate the expression levels of TNF-α with slight up-regulation of expression levels of IKaB, IL-1α, and IL-1β in both infected-AgNPs-treated and AgNPs-treated group. Additionally, the expression levels of IKaB, IL-1α, and IL-1β in AgNPs-treated group were slightly over expressed than that in the infected-AgNPs-treated group, but still significantly lower than that in the infected-untreated group.

**Figure 9:**
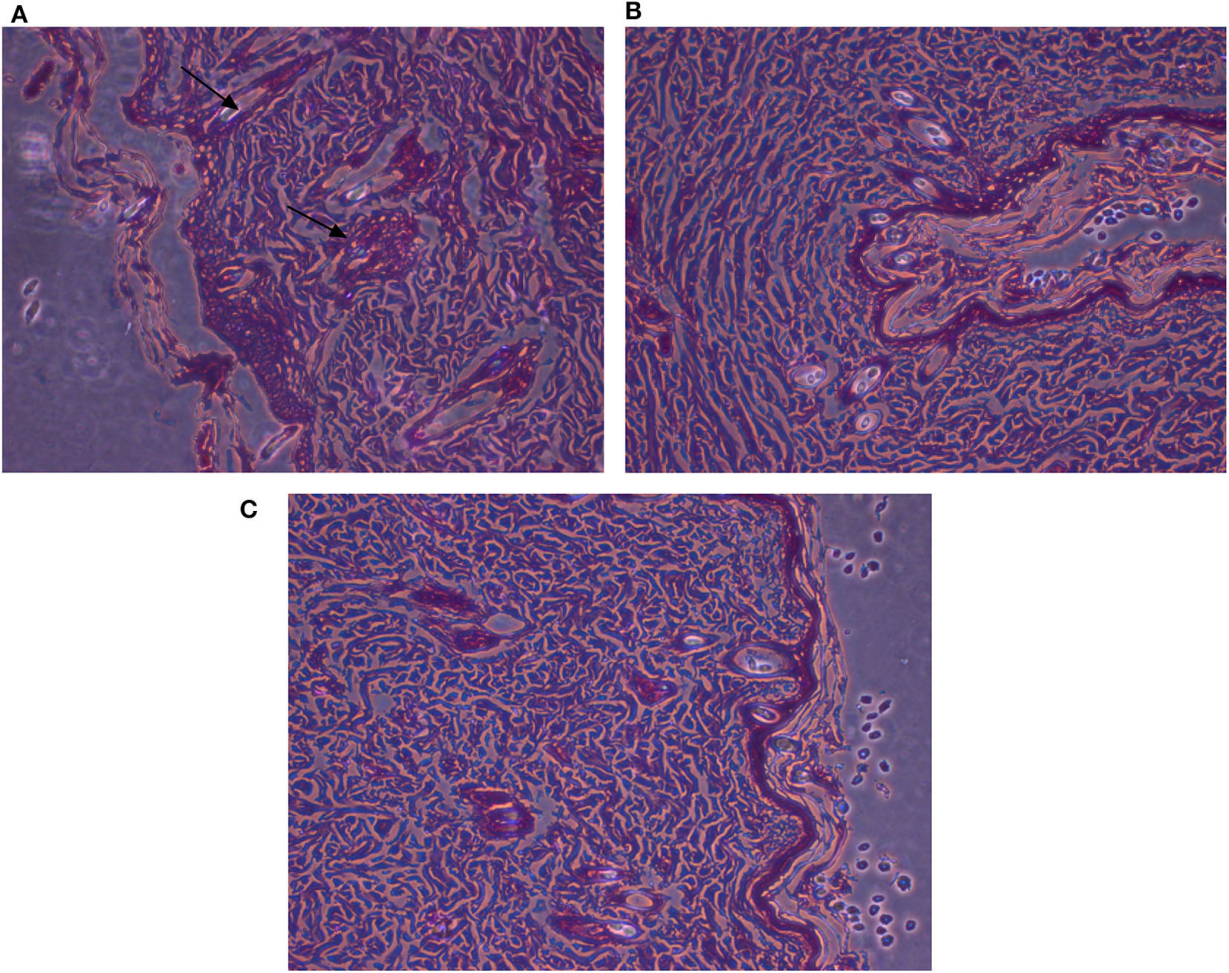
Histopathological examinations of illustrative skin samples (stained with hematoxylin and eosin; ×40) after *P. aeruginosa* infections and upon AgNPs treatment: **(A)** *P. aeruginosa* infected group; **(B)** the non-infected group; **(C)** AgNPs -treated group. *N* = 3. All measurements were done in triplicate and the average was calculated ± standard deviation (*SD*) using GraphPad prism8 software.

#### Histopathologic Investigation

Microscopic examination of animal skin tissues of infected-untreated sections showed distinct morphological changes as displayed in Figure 8B, when compared to the untreated group (Figure 8C), indicating unhealthy cells. A slight thickness in the epidermal layer with more epithelial cells was observed in the infected tissues compared to infected-AgNPs-treated cells. In the infected-AgNPs-treated group (Figure 8D), the distribution of hair follicles was more even than in the bacterial infected tissues. Hemorrhagic areas with infiltrated inflammatory cells (surrounding degenerate or coagulation necrosis hair follicles) and macrophages were observed in bacterial infected tissues with large numbers than that in infected-AgNPs-treated tissues. Hair follicles did not contain hair shafts, and their upper parts were dilated and loaded with keratinized substances. On the other hand, hair follicles disruptions with hemorrhagic areas, infiltrated inflammatory cells (surrounding degenerate or coagulation necrosis hair follicles), and macrophages were observed in bacterial infected tissues with large numbers than that in the AgNPs treated tissue.

## Discussion

Utilization of silver nanoparticles (AgNPs) as a drug delivery system is considered a very propitious approach to improve the advanced antimicrobial systems. These nanoparticles can enhance the antimicrobial properties against bacterial pathogens through a direct adsorption at the cell surface in addition to its oxidative effects (Le Ouay and Stellacci, 2015). Being characterized by their small sizes, this led to facilitated penetration into various living cells resulting in side effects including; cytotoxicity and genotoxicity (Greulich et al., 2011; Chairuangkitti et al., 2013; Liz et al., 2015). However, there is a rising concerns over the biological influences of large-scale use of AgNPs, and the probable risks on human health. Because of this toxicity concerns, safety pattern of the recovered biogenic AgNPs was examined on peripheral blood mononuclear cells, before starting this study. Results showed that AgNPs might be safe to mammalian cells at a concentration of 70 μg/mL with an IC_50_ value of 453 μg/mL. This finding is in agreement with the results recently obtained by a group of researchers who found that the IC_50_ concentrations of chemically synthesized AgNPs on various cell lines, such as mouse fibroblasts, HepG2, and Hela were almost the same, ~7.0 μg/mL (Salomoni et al., 2017).

In medical science fields, the microbial antibiotic resistance is considered as a major issue due to abuse of antibiotics. Another issue associated with the microbial therapy using antibiotic is the emergence of life-threating adverse effects such as hypersensitivity reactions in addition to liver and kidney damages in some cases (Cunha, 2001). In this respect, the evolution of nanotechnology era introduced a promising solution to this problem via AgNPs that showed notable antimicrobial property. Research findings indicated that AgNPs exhibit antimicrobial activity against different Gram-positive and Gram-negative antibiotic-resistant bacteria without significant differences, indicating the broad-spectrum bactericidal effect of AgNPs (Ayala-Nunez et al., 2010). Our findings showed that the growth of all tested bacterial strains was almost completely inhibited after the biosynthesized AgNPs treatment at a dose 500 μg/mL; this finding showed the efficacy usage of AgNPs as antibacterial agent. The microbial activity of AgNPs could be attributed to the release of silver ions; this release could disrupt the bacterial outer membrane, and thus enhance the uptake of Ag^+^ ions and AgNPs leading to reduction of cellular viability (Hakim et al., 2005; Zolghadri et al., 2008; Gnanadhas et al., 2013). For more explanation to the *in vitro* exact mode of AgNPs antibacterial action, we tested the impact of AgNPs against *P. aeruginosa* ultrastructures and multi-drug resistance mechanisms. Results revealed that AgNPs have abelites to disrupt the bacterial outer cell's envelope and accumulate inside treated cells. The antibacterial action of AgNPs could be explained in three different ways: (1) the smallest nanoparticles, with a diameter ranging from 1 to 10 nm become attached to bacterial cellular membrane, and harshly interrupt its proper functioning, (2) then, these particles enter the bacterial membranes, may be via interacting with compounds containing sulfur- and phosphorus-groups such as DNA resulting in membrane damage, and (3) the particles release silver ions which have an additional contribution as bactericidal agents (Morones et al., 2005). On the basis of the preceding discussion, and depending on our findings shown in Figure 1, the recovered AgNPs that had a size of 8–12 nm induced outer membrane breakage of *P. aeruginosa*, thus affected the cellular permeability, and these disruptions, known as “pits,” would result in cell lysis. Interestingly, Similar findings were reported by Kalpana et al. (2019), who indicated that upon treatment of *S. typhimurium* with AgNPs biosynthesized with aqueous extract of *Torreya nucifera*, the TEM analysis showed attachment of the nanoparticles to the bacterial membrane, followed by passing into the cells, disrupting the membrane and leading to leakage of the cellular component as well as cell shrinkage.

Bacterial biofilm is considered one of the main bacterial survival strategies in a diversity of sites in the human body (Dufour et al., 2010). Disruption of biofilm resistance could improve the existing antimicrobials ability against infections. In this context Kalishwaralal et al. reported that AgNPs had antibiofilm activity. They argued that, the use of AgNPs at 100 nM exhibited reduction in the biofilm formation of *P. aeruginosa* and *S. epidermidis* by 95–98% via arresting the production of bacterial exopolysaccharides (Kalishwaralal et al., 2010). In the same context, our results showed that, treating the test strains with MIC 50 dose ranged between 3.5 and 1.7 μg/mL resulted in inhibition of the biofilm formation by a percentage ranged between 52.85 and 79.37%. Indeed, *P. aeruginosa* showed the most resistant biofilm to AgNPs treatment. This finding could be explained by studies of Ahmad et al. (2020). These researchers reported that AgNPs synthesized with *Flacourtia indica* did not show biofilm inhibition activity at doses 15–30 μg/mL, whereas a strong biofilm inhibition was observed at a concentration of 100 μg/mL and *Pseudomonas aeruginosa* (ETPS11) was the most resistant as they appeared as a weak biofilm producer. *P. aeruginosa* biofilm mainly relies on the formation of alginates that represent the major components of the biofilm matrix. They form a shield around the pathogen which prevents penetration of antimicrobials, resulting in a therapeutic failure (Al-Wrafy et al., 2017). Inhibition of *P. aeruginosa* alginate production (even at very low concentration 0.25 mg/ mL) was obtained following treatment with AgNPs could explain the observed antibiofilm activity as one of *P. aeruginosa* multi-resistance mechanisms. In addition to *P. aeruginosa* biofilm and alginate production, formation of pyocyanin is also one of the most potent virulence factors and a major element dictating the progression of biofilm formation and infection (Theerthankar Das et al., 2016). Therefore, our findings related to pyocyanin production inhibition may shed some light about the mechanisms of AgNPs antibacterial effects against *P. aeruginosa* via controlling its multi-resistance elements. In addition, results indicated that AgNPs even at very low concentration (0.25 mg/mL) cause a significant reduction in the pyocyanin production, where ~0.5 mg/mL of Ag NPs showed complete inhibition of pyocyanin formation. Additionally, *P. aeruginosa* β-lactamase production, as well as active efflux of antimicrobials out of the bacterial cells, are extremely important mechanisms developed by pathogens to resist the effect of antibiotics (Lomovskaya and Watkins, 2001). Interestingly, we recorded a marked reduction in the activity and productivity of β-lactamase enzyme, in a concentration-dependent manner. Also, the active efflux of *P. aeruginosa* was completely inhibited at 1 mg/mL of AgNPs, giving greater fluorescence under the UV transilluminator. Similar findings reported by Gupta et al. and Nallathamby et al. who showed that silver nanoparticles could disrupt the MDR kinetics of efflux pump in *P. aeruginosa* isolates (Nallathamby et al., 2010; Gupta et al., 2017). This might be due to termination of the proton gradient exerted by the metal nanoparticles, and hence disruption of the membrane potential, and deterioration of the efflux pump driving force essential for its activity.

In spite of our promising *in vitro* AgNPs antibacterial results, the adverse effects of silver nanoparticles (AgNPs) to humans are our concerns about AgNPs usage. Therefore, we evaluated the AgNPs antibacterial effects using an *in vivo* wound model with monitoring the vital biological parameters in general, wound infections are caused by the surrounding bacteria. These wounds could be infected with various bacterial populations which can be hard to identify, and do not respond to antibiotic treatment, causing chronic non-healing wounds (Westgate et al., 2011). With respect to the *in vivo* safety issues, recent studies have revealed that NPs antimicrobial effects diminish upon interaction with serum proteins. However, findings by Gnanadhasa's et al. conclusively proved that, coating the AgNPs with capping agent could minimize AgNPs binding to the protein of human serum, and could effectively maintain its antibacterial property both in *in vitro* and *in vivo* models (Gnanadhas et al., 2013). This finding is in agreement with our results which showed a slight decrease in the total protein percentage (freely protein) in the serum of AgNPs treated group. We could explain that the algal bio-agent coated substances in AgNPs showed abilities to minimize AgNPs binding to the human serum protein, resulting in a non-significant reduction in the concentrations of serum protein and albumin that could increase the AgNPs cellular uptakes, and the intracellular killing of bacteria. Thus, the capping agents of our biosynthesized AgNPs could play crucial roles in their usage to generate better antibacterial characters with lower toxicity. In addition to protein levels, liver, and kidney functions were monitored to evaluate the safety usage of AgNPs. Several reports have indicated significant alteration in serum and tissue levels of ALT and AST upon AgNPs treatment. In this context, Adeyemi and Adewumi reported a significant increase in the serum and tissue levels of rat ALT and AST after AgNPs treatment (Adeyemi and Adewumi, 2014), which agrees well with results obtained in the present study. In addition, AgNPs treatment did not alter the urea and albumin concentrations, where they maintained normal levels upon treatment and after bacterial infection. On the other hand, Marcato et al. reported a marked increase in the level of urea exerted by AgNPs, leading to less time required for wound healing (Marcato et al., 2015).

Silver nanoparticles might have a role in the granulation tissue formation enhancement and maturation during the wound healing initial stage via reducing the inflammatory cytokines as nitric oxide and prostaglandin E2 (Diniz et al., 2020). The main role of COX enzymes during wound healing was reported by Futagami et al. (2006). These researchers showed an increase in the expression of COX-2 whereas COX-1 was not significantly changed (Futagami et al., 2006). In this context, the possible mechanisms of AgNPs antioxidant activity during wound healing process are not fully explained yet, but few reports tried to clarify their roles in COX enzyme regulations during wound healing process. Our obtained results indicated a significant elevation in COX-2 concentrations in both blood serum and rat skin homogenate. However, upon treatment, these concentrations of COX-2 enzyme decreased from 14.38 to 10.16 ng/ mL without a significant difference with the untreated group. Similar results were obtained by Frankova et al. on fibroblast and keratinocytes which revealed a significant increase in cultures expression of COX-2 in injured skin due to various stimuli such as pro-inflammatory cytokines and lipopolysaccharides (Frankova et al., 2016). Furthermore, Singh et al. indicated that, AgNPs spherical shaped which biosynthesized by *Prunus serrulate* extract exhibited anti-inflammatory action via COX down regulation is a concentration dependent manner (Singh et al., 2017). By using different AgNPs concentrations (1, 10, and 25 μg/ml), the maximum COX2 reduction was recorded at 25 μg/ml AgNPs. In other words, AgNPs wound dressing enhanced the wound healing process *via* cytokine modulation (TNF-α reduction) and lowering of inflammation (Das et al., 2013). These findings are consistent with our results where down-regulation of TNF-α expression and COX-2 reduction were observed with AgNPs treatment. This reduction in the inflammatory mediators could be due to formation of intracellular Ag_2_S *via* induction of H_2_S–synthesizing enzymes in the macrophage that sequestered silver ions liberated form AgNPs, and consequently reducing the inflammation (Gonzalez-Carter et al., 2017). In a similar fashion, down-regulations of TNF-α caused by AgNPs treatment is verified with a slight up-regulation in the expression levels of IKaB, IL-1α, and IL-1β, which are still significantly lower than those in the positive bacterial infected group. This finding was similar to that of Khan et al., who reported a transient increase in the expression levels of IL-1β, IL-6, and TNF-α genes following treatment with nanoparticles (Khan et al., 2013).

Our pathological findings after the *P. aeruginosa* infected wound treatment with AgNPs indicate stimulated histological modifications in the wound healing tissue, as granulation earlier development and maturation. After treatment with AgNPs for 7 days, the tissue structure was restored in the healed region showing stratified epidermis with granular and cornified layers giving evidence about the efficacy of AgNPs in wound healing. Our results are in agreement with others; (Rigo et al., 2013) explained that AgNPs could accelerate granulation tissue formation and maturation, and earlier progress of the primary scar of collagen, and rudimentary cutaneous appendages.

## Conclusions

Results from the current investigation indicated that we have succeeded in the fabrication of a safe and biocompatible AgNPs for antibacterial and wound healing applications without using any solvents or multifaceted preparation methodology. The recovered biogenic AgNPs proved to be non-cytotoxic on PBMc cells, and their antibacterial effects were proved *in vitro* against *Staphylococcus aureus, Streptococcus mutans, Pseudomonas aeruginosa, Salmonella enterica, Escherishia coli*, and *Proteus sp*. (antibiotic resistant bacterial strains). Additionally, the *in vivo* effects were confirmed using *Pseudomonas aeruginosa* infected wound model which highlighted the ability of AgNPs to facilitate the healing process via their antibacterial and anti-inflammatory capacities. However, further preclinical and clinical experiments are required to explain the exact interaction mechanisms between the AgNPs and the immune system to guarantee its effectiveness.

## Data Availability Statement

The raw data supporting the conclusions of this article will be made available by the authors, without undue reservation, to any qualified researcher.

## Ethics Statement

The animal study was reviewed and approved by all *in-vivo* studies were carried out according to guidelines in Tanta University, Faculty of Pharmacy, Tanta, Egypt (REC-FPTU). And the used experimental protocols were approved by the Research Ethical Committee at Faculty of Pharmacy, Egypt under international and institutional guidelines (REC-FPTU).

## Author Contributions

NE-D, MA-E, and LA-M conceived and designed the study and acquired data. All authors contributed to experiments performing, data analysis, and manuscript preparation.

## Conflict of Interest

The authors declare that the research was conducted in the absence of any commercial or financial relationships that could be construed as a potential conflict of interest.
