# Supplementary figures and images for: Biogenically Synthesized Polysaccharides-Capped Silver Nanoparticles: Immunomodulatory and Antibacterial Potentialities Against Resistant Pseudomonas aeruginosa
Source: Front Bioeng Biotechnol. 2020 Jul 21;8:643. doi: 10.3389/fbioe.2020.00643 (PMC7391905; doi:10.3389/fbioe.2020.00643)

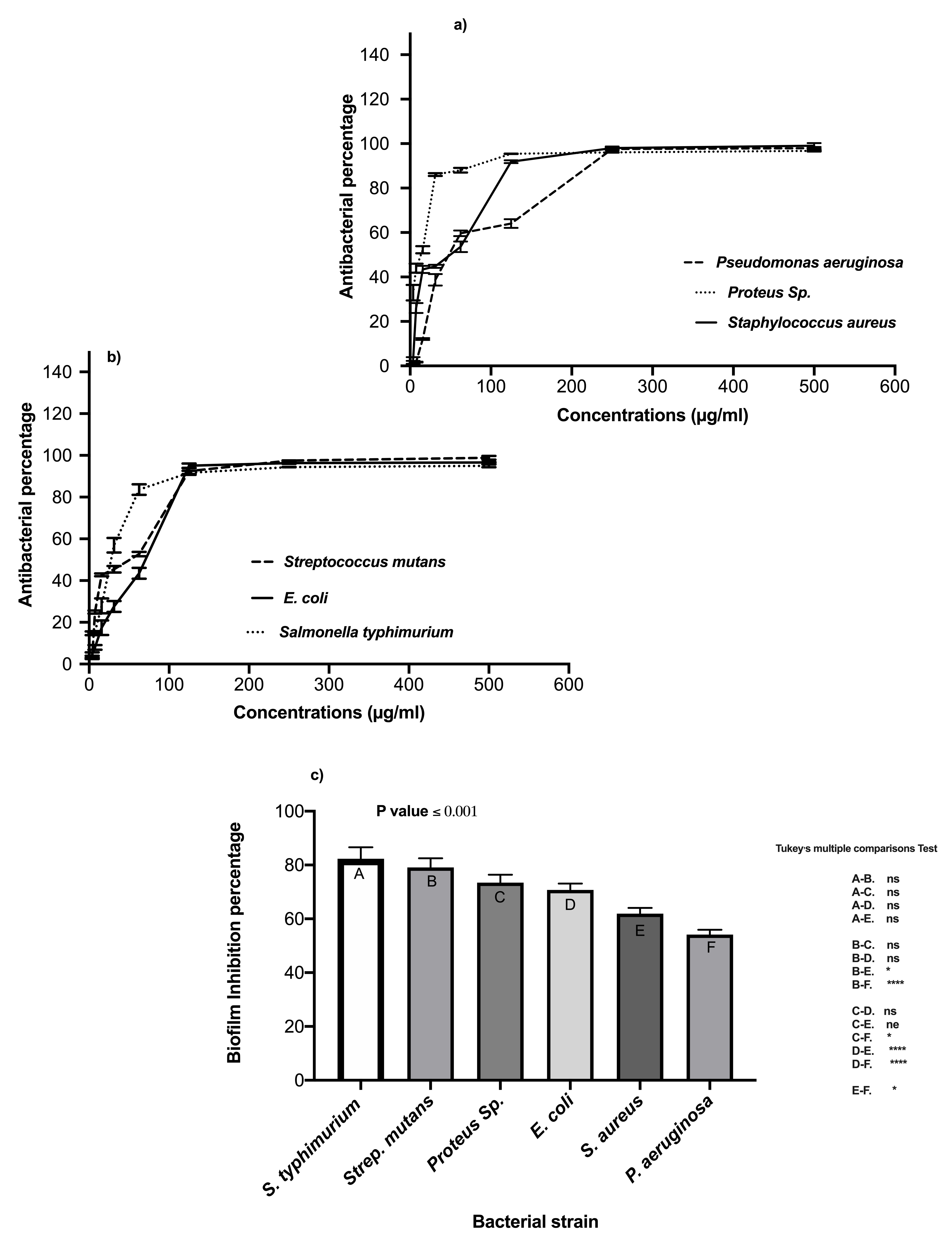

Supplement: Supplementary file 1 [file Image_1.jpeg]
